# Supplementary material for: Comparison of an in-house ‘home-brew’ and commercial ViroSeq integrase genotyping assays on HIV-1 subtype C samples
Source: PLoS One. 2019 Nov 21;14(11):e0224292. doi: 10.1371/journal.pone.0224292 (PMC6871785; doi:10.1371/journal.pone.0224292)

PLOS ONE

Validation of an affordable and sensitive in-house HIV-1 subtype C integrase genotyping assay

--Manuscript Draft--

**Manuscript Number:**	PONE-D-19-15741
**Article Type:**	Research Article
**Full Title:**	Validation of an affordable and sensitive in-house HIV-1 subtype C integrase genotyping assay
**Short Title:**	Affordable INSTI resistance test for HIV-1C
**Corresponding Author:**	Simani Gaseitsiwe, PhD<br>Botswana Harvard AIDS Institute Partnership<br>Gaborone, BOTSWANA
**Keywords:**	dolutegravir; drug resistance, HIV-1; treatment-experienced; Adherence; Botswana
**Abstract:**	Background:<br>Roll-out of Integrase Strand Transfer Inhibitors (INSTIs) such as dolutegravir for HIV combination antiretroviral therapy (cART) in sub-Saharan Africa necessitates the development of affordable HIV drug resistance (HIVDR) assays targeting the Integrase gene. We optimised and evaluated an in-house integrase HIV-1 drug resistance assay (IH-Int) and compared it to a commercially available assay, ViroSeq™ Integrase Genotyping kit (VS-Int) amongst HIV-1 clade C infected individuals.<br><br>Methods:<br>We used 54 plasma samples from treatment naïve participants and one plasma sample from a patient failing INSTI based cART. Specimens were genotyped using both the VS-Int and IH-Int assays. Stanford HIV drug resistance database and the ViroSeq software were used for integrase resistance interpretation. We compared the major and minor resistance mutations, pairwise nucleotide and amino-acid identity, costs and assay time.<br><br>Results:<br>Among 55 specimens tested with IH-Int, 53 (96.4%) successfully amplified compared to 45/55 (81.8%) for the VS-Int assay. The mean nucleotide and amino acid similarity from 33 paired sequences was 99.8% (SD ± 0.30) and 99.8 % (SD ± 0.39) and for the IH-Int and VS-Int assay respectively. The reagent cost/sample were 32 USD and 147 USD for IH-Int and VS-Int assay, respectively. All sequenced samples were confirmed as HIV-1 subtype C.<br><br>Conclusions:<br>The IH-Int assay had a high amplification success rate and high concordance with the commercial assay. It is significantly cheaper compared to the commercial assay. Our assay has the needed specifications for routine monitoring of participants on Dolutegravir based regimens in Botswana.
**Order of Authors:**	Kaelo Keaikitse Seatla
	Wonderful T Choga
	Mompati Mogwele
	Thabo Diphoko
	Dorcas Maruapula
	Lucy Mupfumi
	Rosemary M Musonda
	Christopher F Rowley
	Ava Avalos
	Ishmael Kasvosve
	Sikhulile Moyo

Powered by Editorial Manager® and ProduXion Manager® from Aries Systems Corporation

	Simani Gaseitsiwe
**Additional Information:**	
**Question**	**Response**
**Financial Disclosure**<br><br>Enter a financial disclosure statement that describes the sources of funding for the work included in this submission. Review the submission guidelines for detailed requirements. View published research articles from *PLOS ONE* for specific examples.<br><br>This statement is required for submission and **will appear in the published article** if the submission is accepted. Please make sure it is accurate.<br><br>**Unfunded studies**<br>Enter: *The author(s) received no specific funding for this work.*<br><br>**Funded studies**<br>Enter a statement with the following details:<br>• Initials of the authors who received each award<br>• Grant numbers awarded to each author<br>• The full name of each funder<br>• URL of each funder website<br>• Did the sponsors or funders play any role in the study design, data collection and analysis, decision to publish, or preparation of the manuscript?<br>• **NO** - Include this sentence at the end of your statement: *The funders had no role in study design, data collection and analysis, decision to publish, or preparation of the manuscript.*<br>• **YES** - Specify the role(s) played.<br><br>* typeset	This work was supported through the Sub-Saharan African Network for TB/HIV Research Excellence (SANTHE), a DELTAS Africa Initiative [grant # DEL-15-006]. The DELTAS Africa Initiative is an independent funding scheme of the African Academy of Sciences (AAS)'s Alliance for Accelerating Excellence in Science in Africa (AESA) and supported by the New Partnership for Africa's Development Planning and Coordinating Agency (NEPAD Agency) with funding from the Wellcome Trust [grant # 107752/Z/15/Z] and the UK government. The views expressed in this publication are those of the author(s) and not necessarily those of AAS, NEPAD Agency, Wellcome Trust or the UK government.<br>R.M.M was supported by TESA II which is part of the EDCTP2 programme supported by the European Union (grant number 1051-TESAII-RegNet2015). The views and opinions of authors expressed herein do not necessarily state or reflect those of EDCTP.<br>S.M. was supported in part by the Fogarty International Center and National Institute of Mental Health of the National Institutes of Health under Award Number D43 TW010543.
**Competing Interests**<br><br>Use the instructions below to enter a competing interest statement for this submission. On behalf of all authors, disclose any competing interests that could be perceived to bias this work—acknowledging all financial support and any other relevant financial or non-financial competing interests.	The authors have declared that no competing interests exist.

This statement **will appear in the published article** if the submission is accepted. Please make sure it is accurate. View published research articles from *PLOS ONE* for specific examples.<br><br>**NO authors have competing interests**<br><br>Enter: *The authors have declared that no competing interests exist.*<br><br>**Authors with competing interests**<br><br>Enter competing interest details beginning with this statement:<br><br>*I have read the journal's policy and the authors of this manuscript have the following competing interests: [insert competing interests here]*<br><br>* typeset	
**Ethics Statement**<br><br>Enter an ethics statement for this submission. This statement is required if the study involved:<br><br>• Human participants<br>• Human specimens or tissue<br>• Vertebrate animals or cephalopods<br>• Vertebrate embryos or tissues<br>• Field research<br><br>Write "N/A" if the submission does not require an ethics statement.<br><br>General guidance is provided below. Consult the submission guidelines for detailed instructions. **Make sure that all information entered here is included in the Methods section of the manuscript.**	Ethical clearance for BHP063 and BEAT studies was obtained from the Human Resource Development Committee in Gaborone, Botswana; protocol # HRDC 0638 and HPDME-13/18/1 XI (150) respectively. In addition, BHP063 protocol was approved by the human subjects committee of Harvard T.H Chan School of Public Health (protocol # 20770).

**Format for specific study types**<br><br>**Human Subject Research (involving human participants and/or tissue)**<br>• Give the name of the institutional review board or ethics committee that approved the study<br>• Include the approval number and/or a statement indicating approval of this research<br>• Indicate the form of consent obtained (written/oral) or the reason that consent was not obtained (e.g. the data were analyzed anonymously)<br><br>**Animal Research (involving vertebrate animals, embryos or tissues)**<br>• Provide the name of the Institutional Animal Care and Use Committee (IACUC) or other relevant ethics board that reviewed the study protocol, and indicate whether they approved this research or granted a formal waiver of ethical approval<br>• Include an approval number if one was obtained<br>• If the study involved *non-human primates*, add *additional details* about animal welfare and steps taken to ameliorate suffering<br>• If anesthesia, euthanasia, or any kind of animal sacrifice is part of the study, include briefly which substances and/or methods were applied<br><br>**Field Research**<br><br>Include the following details if this study involves the collection of plant, animal, or other materials from a natural setting:<br>• Field permit number<br>• Name of the institution or relevant body that granted permission	
**Data Availability**<br><br>Authors are required to make all data underlying the findings described fully available, without restriction, and from the time of publication. PLOS allows rare exceptions to address legal and ethical concerns. See the PLOS Data Policy and FAQ for detailed information.	Yes - all data are fully available without restriction

A Data Availability Statement describing where the data can be found is required at submission. Your answers to this question constitute the Data Availability Statement and **will be published in the article**, if accepted.<br><br>**Important:** Stating 'data available on request from the author' is not sufficient. If your data are only available upon request, select 'No' for the first question and explain your exceptional situation in the text box.<br><br>Do the authors confirm that all data underlying the findings described in their manuscript are fully available without restriction?	
**Describe where the data may be found in full sentences. If you are copying our sample text, replace any instances of XXX with the appropriate details.**<br><br>• If the data are **held or will be held in a public repository**, include URLs, accession numbers or DOIs. If this information will only be available after acceptance, indicate this by ticking the box below. For example: *All XXX files are available from the XXX database (accession number(s) XXX, XXX.*).<br>• If the data are all contained **within the manuscript and/or Supporting Information files**, enter the following: *All relevant data are within the manuscript and its Supporting Information files.*<br>• If neither of these applies but you are able to provide **details of access elsewhere**, with or without limitations, please do so. For example:<br><br>*Data cannot be shared publicly because of [XXX]. Data are available from the XXX Institutional Data Access / Ethics Committee (contact via XXX) for researchers who meet the criteria for access to confidential data.*<br><br>*The data underlying the results presented in the study are available from (include the name of the third party*	The 33 pairs of nucleotide sequences obtained in our study were submitted to national center for biotechnology information (NCBI) GenBank and their accession numbers will be availed once they are assigned

and contact information or URL).<br>• This text is appropriate if the data are owned by a third party and authors do not have permission to share the data.<br><br>* typeset	
Additional data availability information:	Tick here if the URLs/accession numbers/DOIs will be available only after acceptance of the manuscript for publication so that we can ensure their inclusion before publication.

Powered by Editorial Manager® and ProduXion Manager® from Aries Systems Corporation

Manuscript
Click here to download Manuscript Seatla et al Assay_FINAL.docx

Validation of an affordable and sensitive in-house HIV-1 subtype C integrase genotyping assay

Kaelo K. Seatla[1,2], Wonderful T. Choga[3], Mompati Mogwele[1,2], Thabo Diphoko[1,2], Dorcas Maruapula[1,2], Lucy Mupfumi[1,2], Rosemary M. Musonda[1,4], Christopher F. Rowley[4], Ava Avalos [1,5,6], Ishmael Kasvosve[2], Sikhulile Moyo[1,4], Simani Gaseitsiwe[1,4,*]

[1]Botswana Harvard AIDS Institute Partnership Gaborone, Botswana

[2]Department of Medical Laboratory Sciences, School of Allied Health Professionals, University of Botswana, Gaborone, Botswana

[3]Division of Human Genetics, Department of Pathology, Faculty of Health Sciences, University of Cape Town, Capetown, South Africa

[4]Department of Immunology & Infectious Diseases, Harvard T.H. Chan School of Public Health, Boston, MA, USA

[5]Careena Centre for Health, Gaborone, Botswana

[6]Ministry of Health and Wellness, Gaborone, Botswana

***Corresponding author**:

Email; sgaseitsiwe@gmail.com (SG)

[#a] Present address: Botswana Harvard AIDS Institute Partnership, Private Bag BO320, Bontleng, Gaborone, Botswana, Phone number +267 390 2671 (office), +267 390 1284 (fax),

Short title; Affordable INSTI resistance test for HIV-1C

ABSTRACT

Background: Roll-out of Integrase Strand Transfer Inhibitors (INSTIs) such as dolutegravir for HIV combination antiretroviral therapy (cART) in sub-Saharan Africa necessitates the development of affordable HIV drug resistance (HIVDR) assays targeting the Integrase gene. We optimised and evaluated an in-house integrase HIV-1 drug resistance assay (IH-Int) and compared it to a commercially available assay, ViroSeq™ Integrase Genotyping kit (VS-Int) amongst HIV-1 clade C infected individuals.

Methods: We used 54 plasma samples from treatment naïve participants and one plasma sample from a patient failing INSTI based cART. Specimens were genotyped using both the VS-Int and IH-Int assays. Stanford HIV drug resistance database and the ViroSeq software were used for integrase resistance interpretation. We compared the major and minor resistance mutations, pairwise nucleotide and amino-acid identity, costs and assay time.

Results: Among 55 specimens tested with IH-Int, 53 (96.4%) successfully amplified compared to 45/55 (81.8%) for the VS-Int assay. The mean nucleotide and amino acid similarity from 33 paired sequences was 99.8% (SD ± 0.30) and 99.8 % (SD ± 0.39) and for the IH-Int and VS-Int assay respectively. The reagent cost/sample were 32 USD and 147 USD for IH-Int and VS-Int assay, respectively. All sequenced samples were confirmed as HIV-1 subtype C.

Conclusions: The IH-Int assay had a high amplification success rate and high concordance with the commercial assay. It is significantly cheaper compared to the commercial assay. Our assay has the needed specifications for routine monitoring of participants on Dolutegravir based regimens in Botswana.

Keywords: dolutegravir, drug resistance, HIV-1, treatment-experienced, adherence, Botswana

Introduction

The global number of people living with HIV (PLWH) has substantially increased over the past two decades; from 16.9 million by the end of 1994 to 36.9 million PLWH by the end of 2017 (1, 2) . Global access to combination antiretroviral therapy (cART) has also increased resulting in a pronounced decline in morbidity and mortality amongst PLWH compared to those who are not on cART (2-4) . This trend is being seen and replicated globally especially with the global scale-up of universal access to cART (2, 5).

Over 20 antiretrovirals drugs (ARVs) spanning 7 drug classes are available in the armament of anti-HIV medicines available to PLWH (6). They are available either as single tablet or a combination of two or more ARVs in single tablet regimens (STR) (6).
Dolutegravir (DTG) is a newer, potent, second generation integrase strand transfer inhibitor (INSTI) that is available as a STR combination with tenofovir disoproxil fumarate (TDF) and lamivudine (3TC) [TLD] at an affordable cost to low and middle income countries (LMICs) such as Botswana (7-10). A majority of countries in sub-Saharan Africa (SSA), a region that is highly burdened by the HIV epidemic will soon have access to TLD (8).

However, there is a rise in pre-treatment and acquired HIV drug resistance (HIVDR) mutations to 'backbone' ARVs such TDF, 3TC and non-nucleoside reverse transcriptase inhibitors (NNRTIs) and this is more pronounced in SSA (11, 12) .This could increase the possibility of placing patients on a 'functional' DTG monotherapy, which can contribute to development of resistance to DTG. A recent modelling study projects that the impact of HIVDR on AIDS deaths, new infections and ART costs will increase in SSA between year 2016-2030 if pre-treatment HIVDR rates are over 10% (13) . The scale-up of TLD will increase the demand for INSTI drug resistance testing. This will be pronounced amongst PLWH previously exposed to first generation INSTIs such as raltegravir (RAL) as is the case in Botswana and other SSA countries.

DTG has a better safety profile, higher genetic barrier to resistance and better clinical efficacy compared to other ARVs such as efavirenz (9, 10, 14, 15) . However, PLWH with prior exposure to RAL that developed a 'Q148 mutant virus' might have reduced

susceptibility to DTG reducing its effectiveness (16, 17) . This has also been demonstrated in case reports from resource limited settings (RLS) in-which individuals with prior RAL exposure who were commenced on DTG combination antiretroviral therapy (cART) developed treatment failure with the selection of the Q148 resistance pathway (18, 19).

Resource rich settings recommend HIV genotypic drug resistance testing (GRT) of the reverse transcriptase (RT) and protease (PR) regions prior to ART initiation and at other time points to guide optimal ARV selections (20, 21) . Due to the low rates of INSTI transmitted drug resistance (TDR) mutations, GRT at the integrase region is not routinely recommended unless clinically indicated such as when patients are experiencing virological failure (VF) while on INSTI cART or if there is a high risk of INSTI TDR mutations (20). In contrast, the World Health Organisation recommends a surveillance and monitoring approach to tackling HIV drug resistance (22). INSTI TDR have been previously reported (23-25) as well as development of INSTI resistance associated mutations (RAMs) with subsequent virological failure amongst ARV treatment experienced individuals in RLS (18, 26).

There are limited options for GRT and most of these are traditionally prohibitively expensive and have been optimised for HIV-1 clade B strain which is not the predominant clade found in SSA. The ViroSeq™ HIV-1 Integrase RUO Genotyping kit (Celera Corporation, USA) (VS-Int) is a commercial assay that detects for HIVDR mutations in the integrase region of *pol* gene. Due to the high cost associated with HIVDR commercial assays, many laboratories have developed less expensive optimised in-house Sanger based (27) GRT assays ("home brews") specific for their most common circulating HIV strains (28-31) .

Botswana, with a predominantly HIV-1C epidemic, introduced DTG into its ART treatment programme in June 2016 and currently relies on commercial assays to test for INSTI HIVDR. Genotypic HIVDR testing typically costs between 140 – 380 USD/ test hindering their use in resource limited settings (RLS) such as in Botswana (32, 33).
Increased access to newer classes of ARVs like DTG in resource limited settings (RLS) where often access to HIVDR testing is limited, raises the possibility of an increase in INSTI RAM rates. There is an urgent need for more frequent VL monitoring and cost effective in-house INSTI HIVDR testing to ensure effectiveness of DTG based ART.

We therefore sought to optimise and evaluate an in-house “home-brew ” integrase drug resistance assay (IH-Int) against a commercial assay, VS-Int, for routine use in Botswana and other countries with a background of HIV-1C infection.

Materials and Methods

Study population, specimen panel and preparations

A total of fifty-five (55) stored plasma samples were used in our assessment. We randomly selected fifty four (54) -80°C stored plasma samples from a previously completed study (BHP063) (34). This study enrolled ART naïve HIV-1 recently infected individuals between January 2012 and December 2015 and it’s described elsewhere (34). The median plasma HIV-1 RNA load (viral loads [VL]) was 4.3 [Q1, Q3 (3.7, 4.9)] log_{10} copies/mL quantified by Abbott m2000sp/Abbott m2000rt (Wiesbaden, Germany).

One (1) well characterised plasma sample collected in 2017 from a highly treatment experienced patient who was enrolled in the Botswana Epidemiological ART Treatment Cohort Study (BEAT) and described elsewhere was included in the analysis as it had many INSTI RAMs determined before by the VS-Int assay (18); The VL was 2.7 log_{10} copies/ml quantified by Cobas TaqMan/Cobas Ampliprep 48 and 96 systems (Roche Diagnostics, Branchburg, USA).

The Abbott and Cobas TaqMan/Cobas Ampliprep assays for VL quantification and HIV drug resistance testing were all performed at the Botswana Harvard HIV Reference Laboratory (BHHRL) in Gaborone, Botswana. BHHRL is a SADCAS ISO 15189 accredited laboratory and maintains certification of the virology assays through Rush University’s virology quality assurance programme.

Ethical considerations

Ethical clearance for BHP063 and BEAT studies was obtained from the Human Resource Development Committee in Gaborone, Botswana; protocol # HRDC 0638 and HPDME-

13/18/1 XI (150) respectively. In addition, BHP063 protocol was approved by the human subjects committee of Harvard T.H Chan School of Public Health (protocol # 20770).

RNA extractions, PCR amplifications and sequencing

VS-Int assay. Genotyping using the VS-Int commercial assay was performed as per the manufacturer's instructions. Briefly, HIV-1 viral RNA was manually isolated from 500µL of participant's plasma by cold pelleting under high-speed centrifugation followed by cell lysis, isopropranolol precipitation and cold ethanol-based re-suspension with elution of 30 µL of HIV RNA. A subsequent one step RT-PCR reaction was performed utilising 10µL of the re-suspended viral RNA. The VS-Int sample preparation and genotyping kits contained all the reagents needed for extraction, reverse transcription polymerase chain reaction (RT-PCR) and sequencing steps that utilise four sequencing primers (Fig 1).

Fig 1. Schematic representation of RT-PCR and sequencing reactions of integrase region using two assays.
Panel A, IH-Int assay RT-PCR and sequencing reaction strategies; Panel B, VS-Int assay RT-PCR and sequencing reaction strategies; IH-Int, in-house " home-brew " integrase drug resistance assay; VS-Int, ViroSeq™ HIV-1 Integrase sample preparation and Genotyping kit (Celera Corporation, USA); aa, amino acids; RT-PCR, reverse transcribed polymerase chain reaction ;kb, kilobase. Source; modified from Abbott ViroSeq™ HIV-1 Integrase genotyping Kit training PowerPoint slides and Wallis C.L et al. /Journal of Virological methods 241 (2017) 41-45

IH-Int assay. Briefly, HIV-1 RNA was automatically extracted from 400µL of plasma by using EZ1 Virus Mini Kit v2.0 (Qiagen, Valencia, CA, USA) cartridges that were loaded on an automated EZ1 Advanced XL (Qiagen) machine. A one step RT-PCR reaction mix was prepared comprising; 0.5µL of Transcriptase Enzyme Roche One Step (Roche, Indianapolis, IN, USA), 7 µL of deionised water (dH_20), 5 µL of Buffer 5X, 2.5 µL Primer mix of 2 µM primer 2F and primer 5R and 10 µL of the extracted viral RNA template for one reaction mix volume totalling 25 µL.

RT-PCR thermal cycling conditions were; one cycle of 50°C for 30 mins, one cycle of 94 °C for 7 minutes, 10 cycles of 94 °C for 10 seconds, 52.5 °C for 30 seconds, 68 °C for 2 minutes, 35 cycles of 94 °C for 10 seconds, 53 °C for 30 seconds, 68 °C for 2 minutes that increased by 10 seconds with each additional cycle with a final extension step of 68 °C for 5 minutes and a hold at 4 °C. A PCR product of about 1300bp was generated that covered the complete HIV-1 integrase region of *pol*.

The primers and reaction mixes components were adapted and modified from experiments conducted on a population largely infected with a clade C virus (28). The PCR thermal cycling conditions were adapted and modified from experiments described elsewhere (34, 35). The RT-PCR products where run on 1% Agarose gel immersed in 1X TBE buffer stained with 3 µL of Ethidium bromide for about 30 mins at 100 volts to verify amplification and correct size of amplicons.

Sequencing

VS-Int assay. Four sequence mixes (forward primers A and B, reverse primers C and D) that came with the VS-Int genotyping kits were each combined with 8µL of the purified, diluted (where necessary) RT-PCR product. Cycle sequencing parameters (25 cycles) were 96 °C for 10 seconds, 50 °C for 5 seconds, 60 °C for 4 minutes and a hold at 4 °C.

Sanger sequencing for VS-Int and IH-Int was performed using an Applied Biosystems 3130xL Genetic Analyser (Applied Biosystems, California, CA, USA).

IH-Int. Column purification of amplicons was performed with QIAquick PCR purification kits (Qiagen, Hilden, Germany). BigDye terminator cycle sequencing ready reaction kit version 3.1 (Applied Biosystems, Carlsbad CA, USA) was used for Sanger sequencing utilising four primers; primer 1F, primer 2F, primer 3R and primer 4R. The cycle sequencing reaction mix contained 3.8 µL of dH_20, 3 µL of Big Dye 5X sequencing buffer, 1 µL Big Dye terminator, 0.2 µL of 10 µM of each sequencing primer and 2 µL of the purified DNA template to make a total reaction volume of 10 µL. Cycle sequencing parameters were the same as for those used for the VS-Int assay.

Purification of cycle sequencing products was done using ZR-96 DNA sequencing clean-up kit (Zymo research, Irvine, CA, USA) according to manufactures instructions.

Sequence, phylogenetic and mutational analysis

Electropherograms obtained were manually assembled and edited using Sequencher® version 5.0 DNA sequence analysis software (Gene Codes Corporation, Ann Arbor, MI, USA) (36) and ViroSeq™ Integrase software v1.0.0 (Celera Corporation, USA) for the IH-Int and VS-Int assays respectively. The generated FASTA files were exported to BioEdit version 7.2.0 software for further analysis (37). Multiple sequences were aligned using ClustalW Multiple alignment programme (38) embedded in BioEdit (37) using the HXB2 (accession number K03455.1) as reference. The integrase sequences generated by the VS-Int and IH-Int methods were then compared for quality assurance and clustering using phylogenetic analysis.

33 pair-matched integrase sequences generated by the VS-Int and IH-Int assays and an HIV-1 reference strain where used to construct phylogenetic trees. Briefly, Bayesian Markov chain Monte Carlo (MCMC) algorithms imbedded in Bayesian evolutionary analysis sampling trees (BEAST) version 1.10.4 and its graphical interface companion programme Bayesian evolutionary analysis utility (BEAUti) 1.10.4 were utilised (39). BEAUti input parameters included the general time reversible (GTR) substitution model with estimated base frequencies and gamma site heterogeneity model utilising four categories with partitions into codon positions switched off. The uncorrelated relaxed clock model with a lognormal relaxed distribution was used and 100,000,000 Markov chain Monte Carlo (MCMC) sampling runs were performed with log parameters recorded every 10,000 (40). The summarised and annotated trees were then visualised using FigTree version 1.4.4 (41) after a burnIn (as states) of 10%, posterior probability limit of 0.90 and above set to be statistically significant in TreeAnnotator version 1.10.4 (39) .

Mutational analysis of sequences was performed using the Stanford HIV drug resistance database algorithm version 8.7 (https://hivdb.stanford.edu/hivdb/by-mutations/). HIV-1 subtype was determined using REGA HIV-1 subtyping tool version 3.0 (http://dbpartners.stanford.edu:8080/RegaSubtyping/stanford-hiv/typingtool/) (42).

Accession numbers

The 33 pairs of nucleotide sequences obtained in our study were submitted to national center for biotechnology information (NCBI) GenBank and their accession numbers will be availed once they are assigned.

Cost comparison

We calculated the reagents costs by analysing a batch of 12 samples used for the IH-Int and VS-Int assay. Estimated costs involved in the IH-Int and VS-Int assay included all stages from extraction, RT-PCR and sequencing.

Statistical analysis

Statistical analysis was performed using STATA version 14 (Stata Corp, College Station, TX, USA. Major and minor HIVDR mutations obtained from both assays were compared as obtained from the Stanford HIV drug resistance database.

Results

Comparison of PCR and sequencing success rates between the VS-Int and IH-Int assay

From the 55 samples, 44 (80%) and 11 (20%) were from female and male participants respectively. Other baseline characteristics are shown in Table 1. 45 (81.8%) of the samples amplified with the VS-Int assay and 53 (96.4%) amplified with the IH-Int assay, Table 2. Of the 10/55 (18.2%) samples that failed amplification with the VS-Int assay, 9/10 (90%) were able to be amplified by the IH-Int assay, Table 3.

Table 1. Baseline demographics and viral load characteristics of 55 HIV-1C infected individuals

Characteristic		n (%)
Sex,	Male	11 (20)
	Female	44 (80)
Age in years Median, (Q1, Q3)		29, (25, 34)
VL >1,000 cps/ml; median log_{10} VL (Q1, Q3) copies/ml		48 (87.3); 4.5 (3.9,4.9)
VL <1,000 cps/ml; median log_{10} VL (Q1, Q3) copies/ml		7 (12.7); 2.7 (2.2,2.7)
VL, viral loads; cps/ml,		

All the study participants were from Botswana. VL, viral load; cps/ml, copies/mL

Table 2. RT-PCR amplification and sequencing success rates of IH-Int and VS-Int assays with further stratification according to viral loads

		IH-Int	VS-Int
Amplification status of participant samples (n, %) n=55; median log_{10} VL (Q1, Q3) cps/ml	Yes;	53 (96.4);<br>4.4 (3.7, 4.9)	45(81.8);<br>4.5 (3.9, 4.9)
	No,	2 (3.8);<br>2.1 & 2.7 log_{10}cps/ml	10 (18.2);<br>3.5 (2.6, 5.0)
Amplification success rate of samples with VL>1,000 cps/ml, n=48, (%); median log_{10} VL (Q1, Q3) cps/ml		48 (100);<br>4.5 (3.9, 4.9)	41 (85.4);<br>4.5 (4.1, 4.9)
Amplification success rate of samples with VL<1,000 cps/ml, n=7, (%); median log_{10} VL (Q1, Q3) cps/ml		5, (71.4);<br>2.7 (2.4, 2.8)	4, (57.1);<br>2.7 (2.6, 2.9)
Sequencing success rate		50/53 (94.3)	38/45(84.4)

The table shows the two assays RT-PCR performance stratified according to viral loads (VL) less than and greater than 1,000 copies/m. VL, viral load; cps/ml, copies/mL; IH-Int, in-house integrase drug resistance assay; VS-Int, ViroSeq™ HIV-1 Integrase sample preparation and Genotyping kit (Celera Corporation, USA); RT-PCR, reverse transcribed polymerase chain reaction.

Table 3. Samples that failed amplification with each assay, VS-Int (table A) and IH-Int (table B) and how they faired when run with a different assay, IH-Int (table A) and VS-Int (table B) and associated viral loads

A	PID	VL log_{10} copies/ mL	Amplifica-tion with VS-Int	Amplifica-tion with IH-Int
1	KKSG41	2.1	N	N
2	KKSG42	2.2	N	Y
3	KKSG43	2.7	N	Y
4	KKSG44	3.2	N	Y
5	KKSG45	3.4	N	Y
6	KKSG46	3.5	N	Y
7	KKSG47	3.7	N	Y
8	KKSG48	4.8	N	Y
9	KKSG49	5.4	N	Y
10	KKSG50	5.7	N	Y

B	PID	VL log_{10} copies/ mL	Amplifica-tion with IH-Int	Amplifica-tion with VS-Int
	KKSG41	2.1	N	N
	KKSG51	2.7	N	Y

Table 3A and B shows the amplification successes of samples that failed amplification with either the VS-Int or IH-Int assay when they were tested with a different assay. PID, participant identification number; N, No; Y, Yes; VL, viral load; IH-Int, in-house integrase drug resistance assay; VS-Int, ViroSeq™ HIV-1 Integrase sample preparation and Genotyping kit (Celera Corporation, USA).

The VS-Int and IH-Int were both a one-step RT-PCR and utilised 4 sequencing primers that covered all of integrase region (Fig 1). Both assays had a similar hands-on and protocol required times, (Fig 2). The VS-Int and IH-Int assays had a sequencing success rate of 38/45 (84.4%) and 50/53 (94.3%), respectively, (Fig 3).

Fig 1. Schematic representation of RT-PCR and sequencing reactions of integrase region using two assays.

Panel A, IH-Int assay RT-PCR and sequencing reaction strategies; Panel B, VS-Int assay RT-PCR and sequencing reaction strategies. IH-Int, in-house “ home-brew ” integrase drug resistance assay; VS-Int, ViroSeq™ HIV-1 Integrase sample preparation and Genotyping kit (Celera Corporation, USA); aa, amino acids; RT-PCR, reverse transcribed polymerase chain reaction ;kb, kilobase. Source; modified from Abbott ViroSeq™ HIV-1 Integrase genotyping Kit training PowerPoint slides and Wallis C.L et al. /Journal of Virological methods 241 (2017) 41-45

Fig 2. Workflow of HIV drug resistance testing using an IH-Int assay (panel A) and VS-Int assay (panel B).

*estimated hands-on time, ** fixed machinery times, [#]Thermocycler times, [+] using Zymogen purification kit;*** on 8 capillary ABI PRISM 3130 xl Genetic Analyser for 12 samples, [±]ViroSeq HIV-1 Integrase Sample Prep Kit 4J94-72, ‡ purification (Exonuclease 1). RT-PCR, reverse transcriptase-polymerase chain reaction; IH-Int, in-house “home-brew” integrase drug resistance assay; VS-Int, ViroSeq™ HIV-1 Integrase sample preparation and Genotyping kit (Celera Corporation, USA).

Fig 3. Differences between amplification and sequencing success rates and cost per test run of two assays.

Viral loads for samples successfully amplified by IH-Int, mean (Q1, Q3); 4.4 (3.7, 4.9), 2.2 copies/ml. Viral loads for samples successfully amplified by VS-Int, mean(Q1,Q3),minimum; 4.4 (3.9, 4.9), 2.6 copies/ml. *S.D = ± 0.30; **S.D= ± 0.39. IH-Int, in-house integrase drug resistance assay; VS-Int, ViroSeq™ HIV-1 Integrase sample preparation and Genotyping kit (Celera Corporation, USA)

Further analysis of both assays on samples with VL greater than 1,000 copies/ml revealed an amplification success rate of 41/48 (85.4%) for the VS-Int and 48/48 (100%) for the IH-Int assay, Table 2. A total of three INSTI mutations were identified by the IH-Int assay and two INSTI mutations were identified by the VS-Int assay, Table 4.

Table 2. RT-PCR amplification and sequencing success rates of IH-Int and VS-Int assays with further stratification according to viral loads

		IH-Int	VS-Int
Amplification status of participant samples (n, %) n=55; median log_{10} VL (Q1, Q3) cps/ml	Yes;	53 (96.4); 4.4 (3.7, 4.9)	45(81.8); 4.5 (3.9, 4.9)
	No,	2 (3.8); 2.1 & 2.7 log_{10}cps/ml	10 (18.2); 3.5 (2.6, 5.0)
Amplification success rate of samples with VL>1,000 cps/ml, n=48, (%); median log_{10} VL (Q1, Q3) cps/ml		48 (100); 4.5 (3.9, 4.9)	41 (85.4); 4.5 (4.1, 4.9)
Amplification success rate of samples with VL<1,000 cps/ml, n=7, (%); median log_{10} VL (Q1, Q3) cps/ml		5, (71.4); 2.7 (2.4, 2.8)	4, (57.1); 2.7 (2.6, 2.9)
Sequencing success rate		50/53 (94.3)	38/45(84.4)

Table shows the two assays RT-PCR performance stratified according to viral loads (VL) less than and greater than 1,000 copies/m. VL, viral load; cps/ml, copies/mL; IH-Int, in-house integrase drug resistance assay; VS-Int, ViroSeq™ HIV-1 Integrase sample preparation and Genotyping kit (Celera Corporation, USA); RT-PCR, reverse transcribed polymerase chain reaction.

Table 4. Comparison of HIV Integrase drug resistance mutations from three patients detected by sanger sequencing using the IH-Int and VS-Int assay and associated viral loads

PID	Source	Viral Load cps/ml	Genotyped region	IH-Int detected mutations	VS-Int detected mutations
				Integrase region positions	Integrase region positions
KKSG19	Plasma	93,154	Integrase	T97A	T97TA
P1	Plasma	4,745	Integrase	E157Q	Nil*
KKSG06	Plasma	515	Integrase	E138K, G140A, S147G, Q148R, T97A	E138K, G140A, S147G, Q148R, T97A

PID, participant identification number; IH-Int, in-house integrase drug resistance assay; VS-Int, ViroSeq™ HIV-1 Integrase sample preparation and Genotyping kit (Celera Corporation, USA); cps/ml, copies/ml; * failed to amplify.

A total of 33 paired sequences revealed a mean amino acid and nucleotide similarity of 99.8 % (SD = 0.39) and 99.8% (SD = 0.30) amongst the two assays fig 4. All sequenced samples were shown to be of a clade C virus by REGA HIV-1 and 2 subtyping tool (43).

The cost of IH-Int were estimated at 32 USD per sample and the VS-Int estimated at 147 USD to run one sample (reagent costs only) and both have similar run times Table 5, Fig 2-3, Fig 4.

Table 5. Comparison between IH-Int assay and commercial VS-Int resistance Assays

	IH-Int assay	VS-Int assay
Description of assay	One step using 4 sequencing primers<br><br>Option of utilising a nested RT-PCR step	One step RT-PCR using 4 sequencing primers<br><br>No option of performing a nested PCR step
Region targeted and positions covered	HIV-1 Integrase region from codons 1-288	HIV-1 Integrase region from codons 1-288
Input sample type	Plasma	Plasma
Volume (µL)	200-400	500
Sample preparation and RNA extraction	Automated EZ1 or manual Qiagen	Manual
Sequencing type	Sanger sequencing using Big Dye chemistry on an ABI PRISM capillary based system	Sanger sequencing using Big Dye chemistry on an ABI PRISM capillary based system
Time to results	17-18 hrs	17-18 hrs
Estimated Costs[+]	32 USD	147 USD *†
Technical skills	High	High
Laboratory set-up	BSL Level 2 or above	BSL Level 2 or above
Weaknesses	Requires costly infrastructure, high technical skills<br><br>Not yet commercially available	Requires costly infrastructure, high technical skills, prohibitively expensive
Strength's	Very affordable	Commercially available, closed, standardized system from extraction, PCR, sequencing and sequence analysis

[+]reagent cost only, *For comparison, HIV-1 PR and RT by ViroSeq® assay is about 155-380 USD cost/test(excluding labour) (33) , † HIV DNA GRT RT is 286 USD and GRT DNA/RNA is 143 USD (32). This table is modified and adapted from table 1, supplementary data of (33) and table 5 of (44). IH-Int, in-house integrase drug resistance assay; VS-Int, ViroSeq™ HIV-1 Integrase sample preparation and Genotyping kit (Celera Corporation, USA);GRT, genotypic resistance test; RT-PCR, reverse transcribed polymerase chain reaction; USD, united states dollars; VL, viral load; PR, protease; RT, reverse transcriptase; BSL, Biosafety level.

Fig 2. Workflow of HIV drug resistance testing using an IH-Int assay (panel A) and VS-Int assay (panel B).

*estimated hands-on time, ** fixed machinery times, #Thermocycler times, + using Zymogen purification kit;*** on 8 capillary ABI PRISM 3130 xl Genetic Analyser for 12 samples, ±ViroSeq HIV-1 Integrase Sample Prep Kit 4J94-72, ‡ purification (Exonuclease 1). RT-PCR, reverse transcriptase-polymerase chain reaction; IH-Int, in-house "home-brew " integrase drug resistance assay; VS-Int, ViroSeq™ HIV-1 Integrase sample preparation and Genotyping kit (Celera Corporation, USA).

Fig 3. Differences between amplification and sequencing success rates and cost per test run of IH-Int and VS-Int assays.
Viral loads for samples successfully amplified by IH-Int, mean (Q1, Q3); 4.4 (3.7, 4.9), 2.2 copies/ml. Viral loads for samples successfully amplified by VS-Int, mean(Q1,Q3),minimum; 4.4 (3.9, 4.9), 2.6 copies/ml. *S.D = ± 0.30; **S.D= ± 0.39. IH-Int, in-house integrase drug resistance assay; VS-Int, ViroSeq™ HIV-1 Integrase sample preparation and Genotyping kit (Celera Corporation, USA).

Fig4. BEAST generated molecular phylogenetic tree of 33 paired sequences generated by population based Sanger sequencing of plasma samples by both VS-Int and IH-Int assays.
All paired sequences had posterior probabilities greater than 99%. BEAST, Bayesian evolutionary analysis; IH-Int, in-house integrase drug resistance assay; VS-Int, ViroSeq™ HIV-1 Integrase sample preparation and Genotyping kit (Celera Corporation, USA).

Discussion

Scale-up of dolutegravir based regimens (DBR) in resource limited settings where HIV-1 clade C is prevalent warrants an urgent need for affordable genotyping HIVDR testing technologies for monitoring INSTI RAMs for patient care and drug resistance surveillance (8, 45) . In this study, we optimised an in-house HIV integrase drug resistance assay and

validated it against a commercial ViroSeq™ HIV-1 Integrase sample preparation and genotyping assay for routine monitoring of drug resistance in Botswana.

Our in-house integrase drug resistance assay was able to amplify 100% (n=48) of patient plasma samples with viral loads greater than 1,000 copies/ml compared to 85.4% (n=48) amplification success rate with the ViroSeq™ HIV-1 Integrase sample preparation and genotyping assay. Similar VS-Int amplification success rates have also been reported by others (15, 30).

In addition, our IH-Int assay was able to amplify 90% of samples that failed amplification with the ViroSeq™ HIV-1 integrase genotyping assay and was also able to detect more INSTI RAMs. The IH-Int assay was able to perform this at approximately a quarter of the cost of the commercial assay.

The higher success rate of the IH-Int could be due to the IH-Int assay having primers that are specific for HIV-1C, while the VS-Int assay primers were designed to cover various HIV-1 subtypes. There is limited published data on the performance of the ViroSeq™ HIV-1 Integrase sample preparation and genotyping assay in HIV-1 non subtype B strains.

Previous studies have shown that the ViroSeq™ Protease-RT assay is less successful with non B HIV-1 subtypes (46, 47). It is most likely that the VS-Int assay has the same limitations as the ViroSeq™ Protease-RT assay.

At 99.8% and 99.8% nucleotide and amino acid identity respectively between the IH-Int and the VS-Int assays, this demonstrates that the sequences generated by the two assays are similar. There is also a lower likelihood that a mutation could be missed by the IH-Int assay that would otherwise be identified by the VS-Int assay. The results of the sequences of the patient with multiple INSTI resistance mutations attest to this good concordance with all the INSTI resistance mutations identified by the VS-Int assay being identified with the IH-Int assay.

The profound cost differences associated with our IH-Int assay in the era of DBR scale-up are encouraging. This high level of RT-PCR success rates and cost savings is similar to what has been traditionally observed with other in-house HIV drug resistance assays (30, 31, 48, 49).

We did not validate our assay amongst non-clade C panel of HIV viruses, as most countries in sub-Saharan Africa have a predominantly clade-C epidemic which may makes our assay applicable to these settings. Further validation of our IH-Int assay using a larger sample size

with multiple users in different laboratory settings in resource limited settings will be required. A better in-house HIVDR assay for patient care would be one that quantifies viral loads, measures ARV drug levels and detects for drug resistance mutations in the integrase, reverse transcriptase and protease region of HIV-1 *pol* gene in one step which could ideally be packaged into a point of care test.

In conclusion, our in-house assay had a high amplification success rate and high concordance with the commercial assay and it is significantly less expensive than the commercial assay.

Our In-house integrase assay has the required specifications to be used in HIV-1 clade C infected individuals for routine monitoring of integrase RAMs in Botswana.

Acknowledgements

We thank our patients and staff of Princess Marina Hospital Infectious Disease Care Clinic (PMH IDCC). We are grateful to the Botswana Harvard HIV Reference Laboratory staff, Botswana Harvard Partnership and Botswana Ministry of Health and Wellness for their collaboration. We also thank Thongbotho Mphoyakgosi, Tshenolo Ntsipe and Segomotso Maphorisa for their outstanding laboratory assistance. We acknowledge Tefo Zongwa and Itseng Bame Montle for their input on the costing analysis. Finally we thank Doreen Ditshwanelo and Naledi T. Gonnye for excellent editorial assistance.

References

1. Burton AH, Mertens TE. Provisional country estimates of prevalent adult human immunodeficiency virus infections as of end 1994: a description of the methods. International journal of epidemiology. 1998;27(1):101-7.
2. UNAIDS. Fact sheet - Latest global and regional statistics on the status of the AIDS epidemic. 18 JULY 2018 [Available from: http://www.unaids.org/sites/default/files/media_asset/UNAIDS_FactSheet_en.pdf.
3. Collaborators GH. Estimates of global, regional, and national incidence, prevalence, and mortality of HIV, 1980-2015: the Global Burden of Disease Study 2015. The lancet HIV. 2016;3(8):e361-e87.
4. Palella FJ, Jr., Delaney KM, Moorman AC, Loveless MO, Fuhrer J, Satten GA, et al. Declining morbidity and mortality among patients with advanced human immunodeficiency virus infection. HIV Outpatient Study Investigators. The New England journal of medicine. 1998;338(13):853-60.
5. Farahani M, Price N, El-Halabi S, Mlaudzi N, Keapoletswe K, Lebelonyane R, et al. Trends and determinants of survival for over 200 000 patients on antiretroviral treatment in the Botswana National Program: 2002-2013. AIDS. 2016;30(3):477-85.
6. AIDSinfo. U.S. Department of Health and Human Services, FDA-Approved HIV Medicines [Available from: https://aidsinfo.nih.gov/understanding-hiv-aids/fact-sheets/21/58/fda-approved-hiv-medicines.
7. UNAIDS C, the Bill & Melinda Gates Foundation, Unitaid, DFID, PEPFAR, USAID, the Global Fund to Fight AIDS, Tuberculosis and, Malaria MLLaAP. New high-quality antiretroviral therapy to be launched in South Africa, Kenya and over 90 low- and middle-income countries at reduced price 2017 [Available from: https://www.unaids.org/en/resources/presscentre/pressreleaseandstatementarchive/2017/september/20170921_TLD.
8. WHO. Dolutegravir (DTG) and the fixed dose combination (FDC) of tenofovir/lamivudine/dolutegravir (TLD) 2018 [Available from: http://www.who.int/hiv/pub/arv/DTG-TLD-arv_briefing_2018.pdf.
9. Cahn P, Pozniak AL, Mingrone H, Shuldyakov A, Brites C, Andrade-Villanueva JF, et al. Dolutegravir versus raltegravir in antiretroviral-experienced, integrase-inhibitor-naive adults with HIV: week 48 results from the randomised, double-blind, non-inferiority SAILING study. Lancet (London, England). 2013;382(9893):700-8.

10. Raffi F, Rachlis A, Stellbrink HJ, Hardy WD, Torti C, Orkin C, et al. Once-daily dolutegravir versus raltegravir in antiretroviral-naive adults with HIV-1 infection: 48 week results from the randomised, double-blind, non-inferiority SPRING-2 study. Lancet (London, England). 2013;381(9868):735-43.

11. Gupta RK, Gregson J, Parkin N, Haile-Selassie H, Tanuri A, Andrade Forero L, et al. HIV-1 drug resistance before initiation or re-initiation of first-line antiretroviral therapy in low-income and middle-income countries: a systematic review and meta-regression analysis. The Lancet Infectious Diseases. 2018;18(3):346-55.

12. TenoRes Study G. Global epidemiology of drug resistance after failure of WHO recommended first-line regimens for adult HIV-1 infection: a multicentre retrospective cohort study. Lancet Infect Dis. 2016;16(5):565-75.

13. Phillips AN, Stover J, Cambiano V, Nakagawa F, Jordan MR, Pillay D, et al. Impact of HIV Drug Resistance on HIV/AIDS-Associated Mortality, New Infections, and Antiretroviral Therapy Program Costs in Sub-Saharan Africa. The Journal of infectious diseases. 2017;215(9):1362-5.

14. Meireles MV, Pascom ARP, Duarte EC, McFarland W. Comparative effectiveness of first-line antiretroviral therapy: results from a large real-world cohort after the implementation of Dolutegravir. Aids. 2019.

15. Walmsley S, Baumgarten A, Berenguer J, Felizarta F, Florence E, Khuong-Josses MA, et al. Brief Report: Dolutegravir Plus Abacavir/Lamivudine for the Treatment of HIV-1 Infection in Antiretroviral Therapy-Naive Patients: Week 96 and Week 144 Results From the SINGLE Randomized Clinical Trial. J Acquir Immune Defic Syndr. 2015;70(5):515-9.

16. Underwood MR, Johns BA, Sato A, Martin JN, Deeks SG, Fujiwara T. The activity of the integrase inhibitor dolutegravir against HIV-1 variants isolated from raltegravir-treated adults. Journal of acquired immune deficiency syndromes (1999). 2012;61(3):297-301.

17. Garrido C, Soriano V, Geretti AM, Zahonero N, Garcia S, Booth C, et al. Resistance associated mutations to dolutegravir (S/GSK1349572) in HIV-infected patients--impact of HIV subtypes and prior raltegravir experience. Antiviral research. 2011;90(3):164-7.

18. Seatla KK, Avalos A, Moyo S, Mine M, Diphoko T, Mosepele M, et al. Four-class drug-resistant HIV-1 subtype C in a treatment experienced individual on dolutegravir-based antiretroviral therapy in Botswana. Aids. 2018;32(13):1899-902.

19. Achieng L, Riedel DJ. Dolutegravir Resistance and Failure in a Kenyan Patient. The Journal of infectious diseases. 2019;219(1):165-7.

20. DHHS. Panel on Antiretroviral Guidelines for Adults and Adolescents. Guidelines for the Use of Antiretroviral Agents in Adults and Adolescents Living with HIV. Department of Health and Human

Services. [Available from: Available at http://www.aidsinfo.nih.gov/ContentFiles/AdultandAdolescentGL.pdf.

21. EACS. European AIDS Clinical Society (EACS) Guidelines version 9.1 October 2018,English [Available from: Available at http://www.eacsociety.org/files/2018_guidelines-9.1-english.pdf.

22. WHO. Global action plan on HIV drug resistance 2017–2021. Geneva: World Health Organization; 2017.Licence: CC BY-NC-SA 3.0 IGO 2017 [Available from: https://www.who.int/hiv/pub/drugresistance/hivdr-action-plan-2017-2021/en/.

23. Cochrane S, Daniel J, Forsyth S, Smit E. First reported case of integrase (R263K, G163R) and reverse transcriptase (M184V)-transmitted drug resistance from a drug-naive patient failing Triumeq. Aids. 2018;32(13):1905-7.

24. Boyd SD, Maldarelli F, Sereti I, Ouedraogo GL, Rehm CA, Boltz V, et al. Transmitted raltegravir resistance in an HIV-1 CRF_AG-infected patient. Antiviral therapy. 2011;16(2):257-61.

25. Young B, Fransen S, Greenberg KS, Thomas A, Martens S, St Clair M, et al. Transmission of integrase strand-transfer inhibitor multidrug-resistant HIV-1: case report and response to raltegravir-containing antiretroviral therapy. Antiviral therapy. 2011;16(2):253-6.

26. Achieng L, Riedel DJ. Dolutegravir Resistance and Failure in a Kenyan Patient. The Journal of infectious diseases. 2018.

27. Sanger F, Nicklen S, Coulson AR. DNA sequencing with chain-terminating inhibitors. Proceedings of the National Academy of Sciences of the United States of America. 1977;74(12):5463-7.

28. Bessong PO, Nwobegahay J. Genetic Analysis of HIV-1 Integrase Sequences from Treatment Naive Individuals in Northeastern South Africa. International journal of molecular sciences. 2013;14(3):5013-24.

29. Hearps AC, Greengrass V, Hoy J, Crowe SM. An HIV-1 integrase genotype assay for the detection of drug resistance mutations. Sexual health. 2009;6(4):305-9.

30. To SW, Chen JH, Wong KH, Chan KC, Ng HM, Wu H, et al. Performance comparison of an in-house integrase genotyping assay versus the ViroSeq Integra48, and study of HIV-1 integrase polymorphisms in Hong Kong. J Clin Virol. 2013;58(1):299-302.

31. Van Laethem K, Schrooten Y, Covens K, Dekeersmaeker N, De Munter P, Van Wijngaerden E, et al. A genotypic assay for the amplification and sequencing of integrase from diverse HIV-1 group M subtypes. J Virol Methods. 2008;153(2):176-81.

32. CMS. Center for Medicare and Medicaid Services, Clinical Laboratory Fee Schedule, Code 87906(Genotype dna/rna hiv) and Code 87901 (Genotype dna hiv reverse t) 2019 [Available from:

https://www.cms.gov/apps/ama/license.asp?file=/Medicare/Medicare-Fee-for-Service-Payment/ClinicalLabFeeSched/Downloads/19CLABQ1.zip.

33. Inzaule SC, Hamers RL, Paredes R, Yang C, Schuurman R, Rinke de Wit TF. The Evolving Landscape of HIV Drug Resistance Diagnostics for Expanding Testing in Resource-Limited Settings. AIDS reviews. 2017;19(4):219-30.

34. Rowley CF, MacLeod IJ, Maruapula D, Lekoko B, Gaseitsiwe S, Mine M, et al. Sharp increase in rates of HIV transmitted drug resistance at antenatal clinics in Botswana demonstrates the need for routine surveillance. The Journal of antimicrobial chemotherapy. 2016;71(5):1361-6.

35. Wallis CL, Papathanasopoulos MA, Lakhi S, Karita E, Kamali A, Kaleebu P, et al. Affordable in-house antiretroviral drug resistance assay with good performance in non-subtype B HIV-1. J Virol Methods. 2010;163(2):505-8.

36. Codes G. Sequencher® version 5.4.6 DNA sequence analysis software, Gene Codes Corporation, Ann Arbor, MI USA [Available from: http://www.genecodes.com/.

37. Hall TA, editor BioEdit: a user-friendly biological sequence alignment editor and analysis program for Windows 95/98/NT. Nucleic acids symposium series; 1999: [London]: Information Retrieval Ltd., c1979-c2000.

38. Thompson JD, Higgins DG, Gibson TJ. CLUSTAL W: improving the sensitivity of progressive multiple sequence alignment through sequence weighting, position-specific gap penalties and weight matrix choice. Nucleic acids research. 1994;22(22):4673-80.

39. Suchard MA, Lemey P, Baele G, Ayres DL, Drummond AJ, Rambaut A. Bayesian phylogenetic and phylodynamic data integration using BEAST 1.10. Virus evolution. 2018;4(1):vey016.

40. Drummond AJ, Ho SY, Phillips MJ, Rambaut A. Relaxed phylogenetics and dating with confidence. PLoS biology. 2006;4(5):e88.

41. Rambaut A. Fig tree v1.4.4 [Available from: http://tree.bio.ed.ac.uk/software/figtree/.

42. Pineda-Pena AC, Faria NR, Imbrechts S, Libin P, Abecasis AB, Deforche K, et al. Automated subtyping of HIV-1 genetic sequences for clinical and surveillance purposes: performance evaluation of the new REGA version 3 and seven other tools. Infection, genetics and evolution : journal of molecular epidemiology and evolutionary genetics in infectious diseases. 2013;19:337-48.

43. Pineda-Peña A-C, Faria NR, Imbrechts S, Libin P, Abecasis AB, Deforche K, et al. Automated subtyping of HIV-1 genetic sequences for clinical and surveillance purposes: performance evaluation of the new REGA version 3 and seven other tools. Infection, Genetics and Evolution. 2013;19:337-48.

44. Inzaule S, Yang C, Kasembeli A, Nafisa L, Okonji J, Oyaro B, et al. Field evaluation of a broadly sensitive HIV-1 in-house genotyping assay for use with both plasma and dried blood spot specimens in a resource-limited country. Journal of clinical microbiology. 2013;51(2):529-39.

45. MoHW. HANDBOOK OF THE BOTSWANA 2016 INTEGRATED HIV CLINICAL CARE GUIDELINES [Available from: https://www.moh.gov.bw/guidelines.html.

46. Beddows S, Galpin S, Kazmi SH, Ashraf A, Johargy A, Frater AJ, et al. Performance of two commercially available sequence-based HIV-1 genotyping systems for the detection of drug resistance against HIV type 1 group M subtypes. Journal of medical virology. 2003;70(3):337-42.

47. Aghokeng AF, Mpoudi-Ngole E, Chia JE, Edoul EM, Delaporte E, Peeters M. High failure rate of the ViroSeq HIV-1 genotyping system for drug resistance testing in Cameroon, a country with broad HIV-1 genetic diversity. Journal of clinical microbiology. 2011;49(4):1635-41.

48. Zhou Z, Wagar N, DeVos JR, Rottinghaus E, Diallo K, Nguyen DB, et al. Optimization of a low cost and broadly sensitive genotyping assay for HIV-1 drug resistance surveillance and monitoring in resource-limited settings. PLoS One. 2011;6(11):e28184.

49. Ammaranond P, Sanguansittianant S, Raju PA, Cunningham P, Horthongkham N. Development of a cost-effective assay for genotyping of HIV-1 non-B subtype for drug resistance. J Virol Methods. 2014;199:102-7.

Figure1 Click here to download Figure Fig1.tif

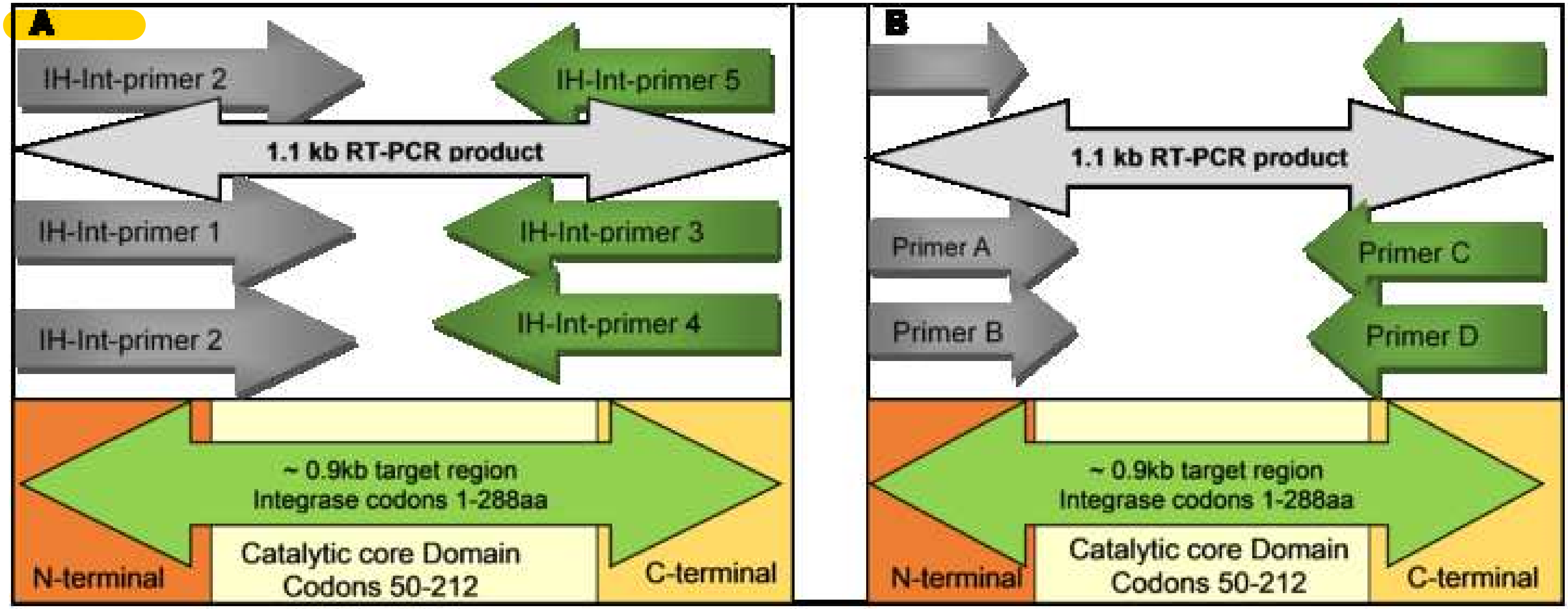

Figure2

Click here to download Figure Fig2.tif

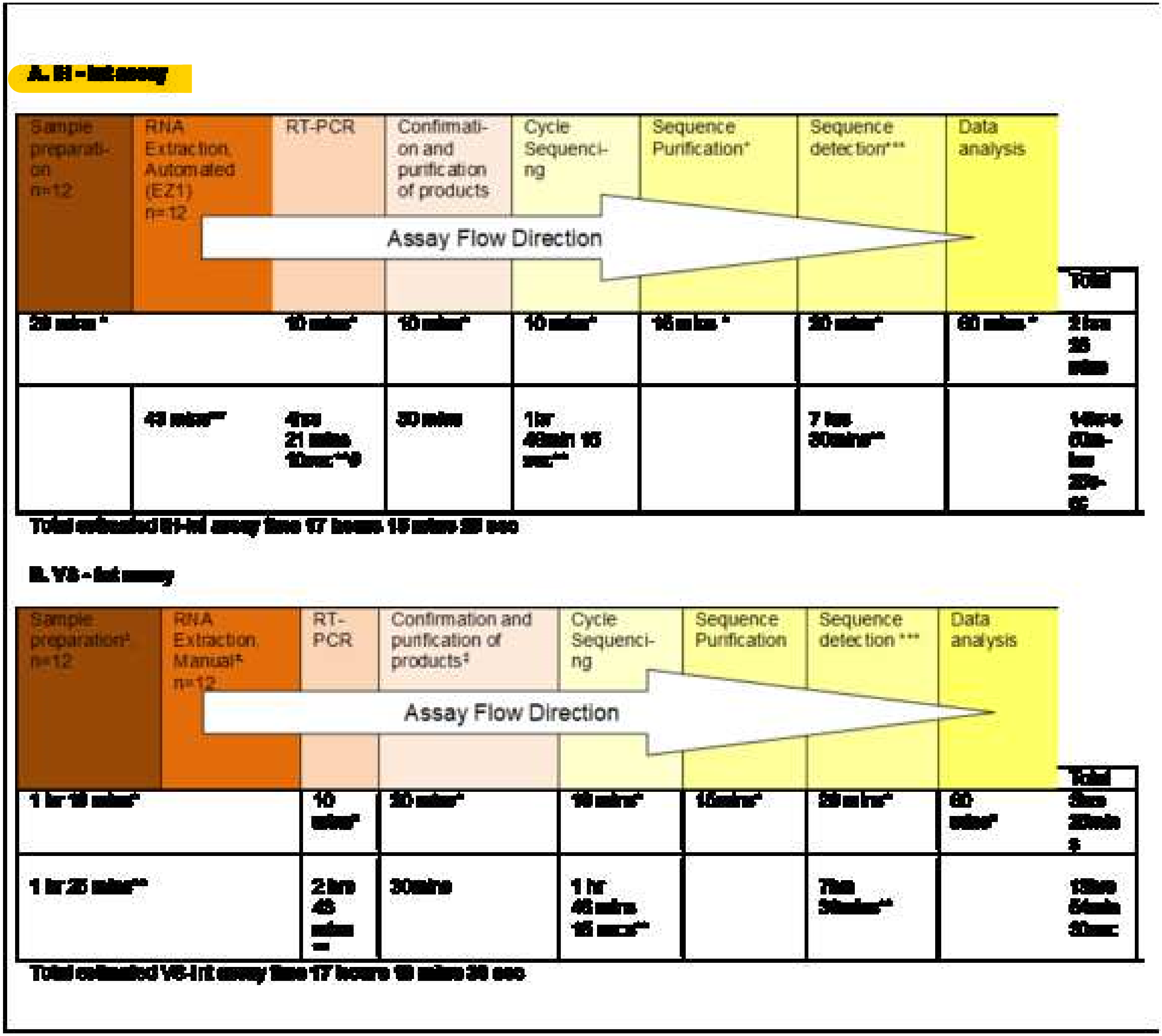

Figure3 Click here to download Figure Fig3.tif

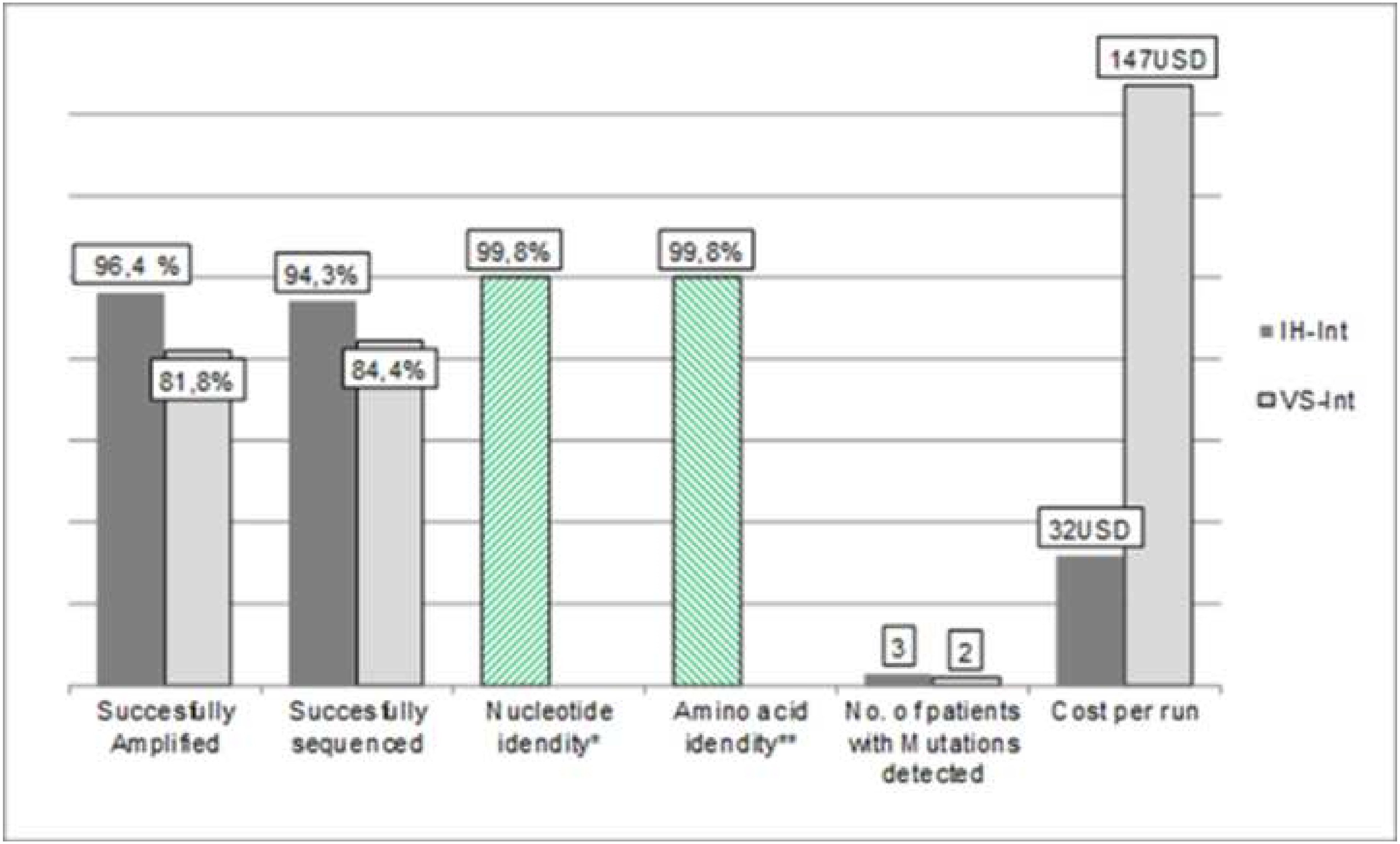

Figure4

Click here to download Figure Fig4.tif

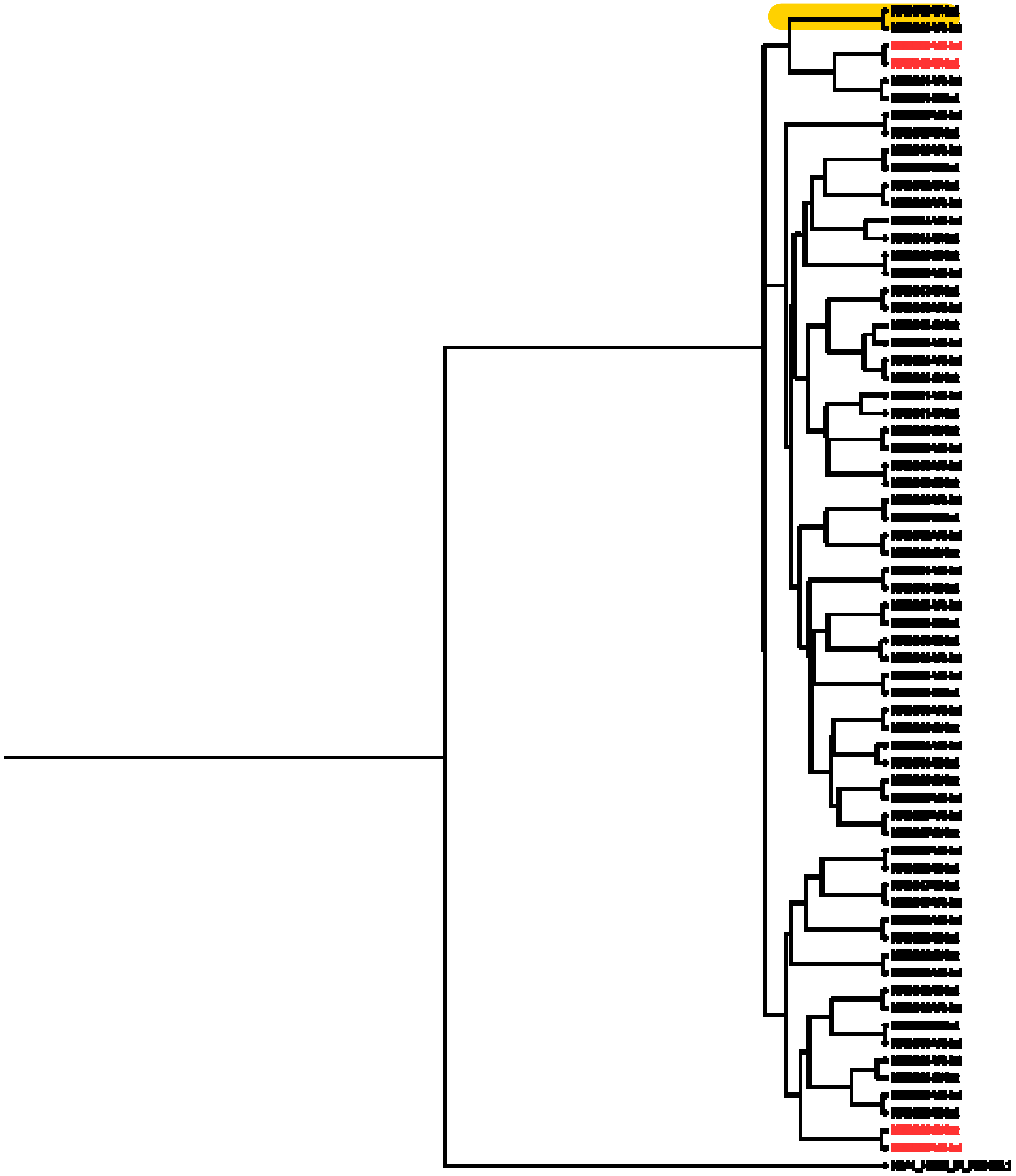

Supplement: Attachment — Submitted filename: PONE-D-19-15741_reviewer_13072019.pdf [file pone.0224292.s001.pdf]
