# Supplementary material for: Comparison of an in-house ‘home-brew’ and commercial ViroSeq integrase genotyping assays on HIV-1 subtype C samples
Source: PLoS One. 2019 Nov 21;14(11):e0224292. doi: 10.1371/journal.pone.0224292 (PMC6871785; doi:10.1371/journal.pone.0224292)
Supplement: Attachment — Submitted filename: PLOS ONE - PONE-D-19-15741_response letter_final.docx [file pone.0224292.s002.docx]

Botswana Harvard AIDS Institute Partnership

Private Bag BO320

Gaborone, Botswana

07^th^ September 2019

Editorial Team

PLOS ONE

Dear Editors

**RE: PLOS ONE PONE-D-19-15741 point-by-point response letter**

We greatly appreciate all the comments from the reviewers, and we believe they have led to the improvement of the manuscript. Here we present our responses to the reviewer comments.

Please do not hesitate to contact us for any further comments.

Sincerely,

Simani Gaseitsiwe, PhD

Laboratory Director at Botswana Harvard AIDS Institute Partnership (BHP)

& Research Associate at Harvard T.H. Chan School of Public Health (HSPH)

Phone: L +267 3902671 ext 2216

M +267 7171 7317

Fax: + 267 390 1284

Reviewer #1: PONE-D-19-15741

Validation of an affordable and sensitive in-house HIV-1 subtype C integrase genotyping assay

This manuscript describes the technical validation of an HIV-1 integrase in-house genotyping assay and compares the cost with its commercial counterpart. Given the recent introduction of integrase inhibitors in many countries, this is a valuable contribution to the scientific community; however there are some limitations to the work.

Major comments:

1. When processing the samples, were repeat testing for PCR and/or sequencing allowed or was only 1 attempt to obtain a result allowed. This should be included in the materials and methods. Especially the poor sequencing success rate is unexpected for samples with a suitable PCR product

Response:

Thank you for pointing this out. Only one attempt to obtain results was allowed, there was no multiple testing on the same sample. We have included in the material and methods section, line 182 the following; “For both assays, only one attempt to obtain PCR or sequencing result was used, no repeat testing of samples using both assays were allowed.”

2. At what level was mixed based calling performed? 20%, 25%, were the same rule applied for VS-Int and IH-Int since different sequence analysis software was used.

Response:

Thank you for pointing this out. We had attempted to keep the VS-INT manufacturer’s instruction. In the revised manuscript, in order to avoid bias we have reanalysed sequences using Sequencher 5.0 for both assays-the VS-Int and IH int. This re-analysis did not have any impact in the results. We have modified and included in the materials and methods section, under sub-heading Sequence, phylogenetic and mutational analysis from line 215 the following; “Electropherograms obtained were manually assembled and edited using Sequencher® version 5.0 DNA sequence analysis software (Gene Codes Corporation, Ann Arbor, MI, USA) (36) for both the IH-Int and VS-Int assays respectively. The Sequencher assembly parameters used were a minimum match percentage of 85% and a minimum overlap of 20 base pairs for sequences derived by both the IH-Int and VS-Int assays”.

3. Why were there only 33 paired sequences available, is this caused by the inability to obtain good sequence quality for a number of specimens using VS-Int

Response:

We have included the following in line 307; “From the 50 sequences obtained by the IH-Int assay and 38 sequences obtained by the VS-Int assay, 33 paired sequences were identified. The low number of paired sequences was due to failures in amplification and sequencing by the VS-Int assay”.

As shown in Table 2 the amplification success rate for VS-Int was 82% as compared to 96% for the IH-Int, and for samples with viral load > 1000 copies/mL, the amplification success rate for VS-Int was 85% and 100% for the IH-Int assay.

4. Table 4, I don’t think it is correct to say that VS-Int did not pick up the mutation (nil) because there was no PCR product available for this specimen. I would rather change nil to N/A or even remove it from the table

Response:

Thank you for pointing this out, in line 305 table 4 row 3, we have changed ‘nil’ to “N/A”.

5. I think the statement on line 359 regarding the IH assay’s ability to detect more mutations is incorrect. If sample P1 failed to amplify using VS, one cannot make a statement regarding the inability to detect that mutation. For KKSG19 the mutation T97A was detected by both assays, albeit it being a mix for VS. In addition, based on the limited number of specimens with DRMs, no strong statement should be made regarding the ability to detect mutations.

Response:

Thank you for the comment. In line 353, we have modified the first sentence to reflect this clearly, we have deleted “…and was able to detect more INSTI RAMs…”. The sentence in line 353 now reads; “In addition, our IH-Int assay was able to amplify 9/10 (90%) of samples that failed amplification with the ViroSeq™ HIV-1 integrase genotyping assay”.

6. Clarify statement on line 371-372: “there is also a lower… by the VS Int assay.

Response:

In line 365, we have deleted the sentence “There is also a lower likelihood that a mutation could be missed by the IH-Int assay that would otherwise be identified by the VS-Int assay”.

7. Table 5 includes the option of utilising a nested RT-PCR step for IH-Int. Was nested PCR performed for any of the samples, if yes, for how many? And how did this impact the success rate of the IH assay?

Response:

Thank you for highlighting this point. Table 5, line 316, second column under heading “IH-Int assay”, we have modified as follows; “Has option of utilising a nested RT-PCR step available but this wasn’t used in our comparison”.

8. Were reproducibility and specificity assessed for the IH assay. I think it is important to show that the same results (for PCR and sequencing) can be obtained when the same sample is repeated. If not yet done, reproducibility should be assessed and results should be included in the manuscript.

Response:

The tittle of our manuscript has been changed from “Validation of an affordable and sensitive in-house HIV-1 subtype C integrase genotyping assay” to “Comparison of an in-house ‘home-brew’ and commercial ViroSeq integrase genotyping assays on HIV-1 subtype C samples”.

In addition, we have added into the limitations under the discussion line 374 the following; “Our sample size and volumes did not permit an evaluation of reproducibility and specificity hence they were not assessed but are in the future directions.”

9. I understand that samples with InSTI mutations are difficult to find, but there have been reports from more than 1 patient from Botswana with InSTI mutations, are those samples available? If not, do you have access to EQA material with InSTI mutations? The inclusion of more samples with InSTI mutations would significantly improve the value of the manuscript

Response:

Yes the reports of patients with InSTI mutations are correct. There are a few numbers of patients with InSTI mutations in Botswana but were not all part of this sample at the time of this study. We have 1 case of multi-class HIV DR mutations already included in this study. We plan further evaluations beyond subtype C and your suggestions are appreciated. We have since modified the title to reflect this as a comparison study as opposed to a validation.

Minor comments:
1. Line 41-42: “…and for IH-int and VS-Int assay respectively” seems to be out of place in this sentence

Response:

We have modified the sentence and deleted the “…and…”. Line 45 now reads thus; “The mean nucleotide and amino acid similarity from 33 paired sequences was 99.8% (SD ± 0.30) and 99.8 % (SD ± 0.39) for the IH-Int and VS-Int assay respectively.”

2. Table 1: second last line looks like an error

Response:

In table 1,line 263, we have deleted the second last line.

3. Table 2 on page 13 seems to be a repeat from table 2 on page 10

Response:

This is correct, we have deleted table 2 on page 13.

4. I don’t see the value of figure 1 as primers seem to cover the same target of interest (both aa 1-288), however the position of the primers seems to be different between the assays.

Response:

We have removed fig 1 from the manuscript

5. Figure 2 is illegible

Response:

We have included the same fig 2 but with better resolution to improve illegibility. Figure 2 has now been renamed to figure1. In the former fig 2,now fig 1, we have deleted “Including nested PCR total time 21 hours 46 mins 35 sec”in panel A and “Total (excluding nested step)” caption in panel last right column.

Reviewer #2: Thank you for the opportunity to review this very important manuscript.
Major comments:

1. The validation appears to be incomplete as a number of experiments which are usually part of a start validation exercise are missing. The following experiments would have added more value to the validation:
i) Validation should ideally include a set of sample on know genotypes - preferably EQA samples or samples genotyped by another method other than the methods being compared.

Response:

Thank you for pointing this out. The tittle of our manuscript has been changed from “Validation of an affordable and sensitive in-house HIV-1 subtype C integrase genotyping assay” to “Comparison of an in-house ‘home-brew’ and commercial ViroSeq integrase genotyping assays on HIV-1 subtype C samples”.

In addition, we have added into the limitations under the discussion line 374 the following; “Our sample size and volumes did not permit an evaluation of reproducibility and specificity hence they were not assessed but are in the future directions.”

ii) Serially diluting a high viral load sample 3-5 samples (each dilution having at least 3 replicates) to determine the dynamic range of amplification of the method being validated (and that of the comparator) - this is ideal done using EQA samples/panel of samples.

Response:

The tittle of our manuscript has been changed from “Validation of an affordable and sensitive in-house HIV-1 subtype C integrase genotyping assay” to “Comparison of an in-house ‘home-brew’ and commercial ViroSeq integrase genotyping assays on HIV-1 subtype C samples”.

In addition, we have added into the limitations under the discussion line 374 the following; “Our sample size and volumes did not permit an evaluation of reproducibility and specificity hence they were not assessed but are in the future directions.”

iii). Testing the replicates on different days or in different runs (The report does not indicated over how many runs and days the experiments were done).
Otherwise, this is just a head to head comparison of two methods.

Response:

Thank you for highlighting this. This is correct, indeed the tittle of our manuscript has been changed from “Validation of an affordable and sensitive in-house HIV-1 subtype C integrase genotyping assay” to “Comparison of an in-house ‘home-brew’ and commercial ViroSeq integrase genotyping assays on HIV-1 subtype C samples”.

We have added into the limitations under the discussion section about our sample size and volume of samples that did not permit an evaluation of reproducibility and specificity were not assed but are in the future directions.

2. The performance of the two assays should be measure with the percentage of genotypes obtained from the initial samples tested. The percentage of samples amplified is an interim result. So the main results in terms of the performances of the two assays should be 50/55 (91%) vs 38/55 (69%).

Response:

Thank you for pointing this out. This has been corrected in Table 2 line 264. The sequencing success rate has been changed to 50/55 (90.9) for the IH-Int and 38/55 (69.1) for the VS-Int

Minor comments
1. Since the primers and the method used for the in-house method were adopted and modified from a previously published paper, the authors should provide details of the new primers including sequences and HXB2 position.

Response:

Under materials and methods, sub heading RNA extractions, PCR amplifications and sequencing, from line 170, we have modified to include the following; “A one step RT-PCR reaction mix was prepared comprising; 0.5µL of Transcriptase Enzyme Roche One Step (Roche, Indianapolis, IN, USA), 7 µL of deionised water (dH20), 5 µL of Buffer 5X, 2.5 µL primer mix of 2 µM INFORI (5' GGA ATC ATT CAA GCA CAA CCA GA 3' nucleotide positions relative to HXB2 4059-4081) and INREV-1 ( 5’-TCT CCT GTA TGC AGA CCC CAA TAT-3’ 3' nucleotides positions relative to HXB2 5244-5267) and 10 µL of the extracted viral RNA template for one reaction mix volume totalling 25 µL.”

In addition, the sequencing primers used for the IH-Int assay have been included in line 199 and reads thus; “IH-Int. Column purification of amplicons was performed with QIAquick PCR purification kits (Qiagen, Hilden, Germany). BigDye terminator cycle sequencing ready reaction kit version 3.1 (Applied Biosystems, Carlsbad CA, USA) was used for Sanger sequencing utilising four primers; HIV+4141 ( 5' TCT ACC TGG CAT GGG TAC CA 3' nucleotide positions relative to HXB2 4141-4160), INFORI ( 5' GGA ATC ATT CAA GCA CAA CCA GA 3' nucleotide positions relative to HXB2 4059 -4081), INREVII (5' CCT AGT GGG ATG TGT ACT TCT GA 3' nucleotide positions relative to HXB2 5197-5219 and IN4764AS (5' CCATTTGTACTGCTGTCTTAA 3’ 4764-4744)”.

2. The phylogenetic tree, figure 4, should contain all 88 sequences generated from sequencing using the two methods (50 from the In-house method and 38 from VS.

Response:

Thank you for highlighting this. The phylogenetic tree figure 4, is now figure 3. We have included a phylogenetic tree including all the sequences and a reference strain.

In addition, this has been included in the manuscript from line 222; “The 88 integrase sequences generated by the VS-Int and IH-Int methods were then compared for quality assurance and clustering using molecular phylogenetic analysis by maximum likelihood method in MEGA 7 software (39). The Phylogenetic tree was constructed using Tamura-Nei substitution model with gamma distribution rates among sites and inferred from 1000 bootstrap replicates (fig3) (40)”.

3. Figure 3 is not clear

Response:

Fig 3 has been renamed fig 2. The figure has been reattached with a better resolution.

4.All the sequences above should also be submitted to Genbank instead of just the 33 pairs.

Response:

The 33 paired sequences have been submitted to Genbank and the accession numbers are available and are included in line 247 under materials and methods and subheading accession numbers; “The 33 pairs of nucleotide sequences obtained in our study were submitted to national center for biotechnology information (NCBI) GenBank and their accession numbers are MN037428 to MN037493”.

We are in process of submitting to GenBank the remaining sequences.

5. Please the comments on the attached file for the rest of the comments.

Response:

In addition, during revision of table 2 with our data set, we identified some minor corrections; under the IH-Int column, row 3, Q3 is 4.8 not 4.9. Row 4, percentage is 3.6% not 3.8%, and row 5 Q1;Q3 are 2.6 and 2.7 instead of 2.4,2.8.

Under the VS-Int column, row 2 Q3 is 4.8 not 4.9, row 3 Q1;Q2 is 2.8,4.5 not 2.6,5.0 and row 5 Q1,Q3 is 2.7,2.8 not 2.6,2.9.
